# Repurposing Dimetridazole and Ribavirin to disarm *Pseudomonas aeruginosa* virulence by targeting the quorum sensing system

**DOI:** 10.3389/fmicb.2022.978502

**Published:** 2022-08-15

**Authors:** Yang Yuan, Xiting Yang, Qianglin Zeng, Heyue Li, Ruyi Fu, Lianming Du, Wei Liu, Yamei Zhang, Xikun Zhou, Yiwen Chu, Xiuyue Zhang, Kelei Zhao

**Affiliations:** ^1^Key Laboratory of Bio-resources and Eco-environment, Ministry of Education, College of Life Sciences, Sichuan University, Chengdu, Sichuan, China; ^2^Antibiotics Research and Re-evaluation Key Laboratory of Sichuan Province, School of Pharmacy, Affiliated Hospital/Clinical College of Chengdu University, Chengdu, Sichuan, China; ^3^State Key Laboratory of Biotherapy and Cancer Center, West China Hospital, West China Medical School, Sichuan University, and Collaborative Innovation Center for Biotherapy, Chengdu, Sichuan, China

**Keywords:** *Pseudomonas aeruginosa*, Dimetridazole, Ribavirin, functional profiling, antivirulence, quorum sensing

## Abstract

*Pseudomonas aeruginosa* relies on its complex cellular regulatory network to produce a series of virulence factors and to cause various acute and chronic infections in a wide range of hosts. Compared with traditional antibiotics which frequently accompany with widespread antibiotic resistance, crippling the virulence system of bacteria is expected to be a promising anti-infective strategy. In this study, Dimetridazole and Ribavirin, which had poor antibacterial activities on *P. aeruginosa* reference isolate PAO1 in nutrient medium but significantly inhibited the growth of *P. aeruginosa* PAO1 in M9-adenosine, were selected from 40 marketed compounds with similar core structure (furan, benzofuran, or flavonoids) to the acyl-homoserine lactone signals of *P. aeruginosa* quorum sensing (QS) system. The production of QS-controlled proteases, pyocyanin, and biofilm formation of *P. aeruginosa* PAO1 and the clinical isolates were significantly decreased by the presence of Dimetridazole or Ribavirin. Correspondingly, the majority of QS-activated genes in *P. aeruginosa*, including the key regulatory genes *lasR*, *rhlR*, and *pqsR* and their downstream genes, were significantly inhibited by Ribavirin or Dimetridazole, as determined by RNA-sequencing and quantitative PCR. Furthermore, the susceptibilities of drug-resistant *P. aeruginosa* isolates to polymyxin B, meropenem, and kanamycin were remarkably promoted by the synergistic application of Dimetridazole or Ribavirin. Finally, the treatment of Ribavirin or Dimetridazole effectively protected *Caenorhabditis elegans* and mice from *P. aeruginosa* infection. In conclusion, this study reports the antivirulence potentials of Dimetridazole and Ribavirin on *P. aeruginosa* and provides structural basis and methodological reference for the development of anti-pseudomonal drugs.

## Introduction

Since the discovery of penicillin by Alexander Fleming in 1928, antibiotics have saved countless lives as a first-line treatment against bacterial infections for nearly 90 years (Czaplewski et al., 2016; Hutchings et al., 2019). However, the widespread use and abuse of antibiotics have greatly facilitated the emergence of drug-resistant and multi-resistant pathogens (Dickey et al., 2017; Nadeem et al., 2020). Drug-resistant pathogen-related infections cause nearly 700,000 deaths per year worldwide, and it is projected that there would be more than 10 million deaths in 2050, far higher than the 8.2 million deaths caused by cancer (Tagliabue and Rappuoli, 2018). Traditional antibiotics usually inhibit the biological processes (such as cell wall synthesis, DNA replication, RNA transcription, and protein synthesis) of bacterial pathogens (Lu et al., 2022). However, it is well recognized that such high selection pressures significantly contribute to the rapid emergence and prosperity of antibiotic-resistant bacterial strains, and finally lead to the failure of clinical therapy and urgent need for the development of novel anti-infectious drugs (Lu et al., 2022).

*Pseudomonas aeruginosa* is a Gram-negative bacterium that exists in a wide range of natural and clinical environments (Kalia et al., 2019). It is also an important opportunistic pathogen that often causes hospital acquired infections of immunocompromised patients with cystic fibrosis, burns, surgical wounds, urinary tract or other acute, and chronic diseases (Zhao et al., 2019; Kumar et al., 2021). The relatively large genome size and highly complex regulatory network confer *P. aeruginosa* significant congenital and acquired (e.g., drug resistance) growth advantages to colonize different habitats (Stover et al., 2000; Balasubramanian et al., 2013; Kumar et al., 2021). It has been well-accepted that quorum sensing (QS), which describes the process of signal molecule-mediated cell–cell communication, plays a vital role in population proliferation, the development of virulence and resistance, and immune evasion of *P. aeruginosa* in the host (Moradali et al., 2017; Song et al., 2019; Zhao et al., 2019). The QS system of *P. aeruginosa* is mainly composed of *las-*, *rhl-and pqs-*system with *las* sits atop of the other two (Lee and Zhang, 2015). The *las-and rhl-*QS systems have complete self-induced regulatory system including the signal molecule synthesis protein (LasI/RhlI) and regulatory protein (LasR/RhlR; Smith et al., 2002; Balasubramanian et al., 2013; Lu et al., 2022). The acyl-homoserine lactone (AHL) signal molecules *N*-(3-oxo-dodecanoyl)-*L*-homoserine lactone (3-oxo-C12-HSL)/*N*-butanoyl-*L*-homoserine lactone (C4-HSL) synthesized by LasI/RhlI can bind to LasR/RhlR and regulate the expression of downstream functional genes (Jimenez et al., 2012; Balasubramanian et al., 2013). The regulatory protein PqsR of the *pqs-*system receives signal molecules produced by other pathways, for example, *Pseudomonas* Quinolone signal (PQS) to achieve complete functional regulation (Jimenez et al., 2012; Balasubramanian et al., 2013). The QS system of *P. aeruginosa* regulated the expression of at least 384 genes, including the genes encoding the majority of extracellular virulence determinants, such as elastase, exotoxinqq, rhamnolipids, cyanide, and pyocyanin (Schuster et al., 2003; Lequette et al., 2006; Diggle et al., 2007; Guo et al., 2013).

Among the recently emerging therapeutic strategies, disarming the virulence of bacteria but not directly killing them has been considered to be a promising alternative to combat bacteria (Fleitas Martínez et al., 2019). Antivirulence therapy is envisaged to inhibit the specific functions required by pathogens to cause infection, such as the production of toxins, cytolytic cytokines, proteases, and other mechanisms that can cause host tissue damage (Clatworthy et al., 2007; Escaich, 2010). Theoretically, antivirulence drugs bring low selection pressure for the growth of bacterial pathogens and thus might be a potential development direction of anti-infective drugs (Allen et al., 2014; Gerdt and Blackwell, 2014). As one of the bacterial species with well-characterized QS system, *P. aeruginosa* is frequently used as a model species in the screening and functional verification of antivirulence drugs, or QS inhibitors (Paczkowski et al., 2017; Schütz and Empting, 2018; Soukarieh et al., 2018). For example, quercetin (a natural flavonol commonly found in vegetables and fruits) and 6-gingerol (a pungent oil of fresh ginger) have been found to bind LasR and RhlR and inhibit the production of pyocyanin, protease, and elastase and biofilm formation of *P. aeruginosa* (Kim et al., 2015; Ouyang et al., 2016). Some marketed antibiotics, such as azithromycin, tobramycin, ciprofloxacin, and doxycycline have also been reported to function as QS inhibitors and achieve unexpected therapeutic effect against *P. aeruginosa* infection (Fonseca et al., 2004; Babić et al., 2010; Husain and Ahmad, 2013; Imperi et al., 2014; Gupta et al., 2015; Kumar et al., 2021). These findings give us a hint that there might be kinds of compounds with antivirulence potential among the known natural products and marketed drugs, but the function of which still remain unexplored.

Hence, in this study, we tested the antivirulence activity of a series of known compounds, which have similar core structure (furan, benzofuran, or flavonoids) to the AHL signals of *P. aeruginosa* but were not developed for antibacterial use. Finally, we found that the broad-spectrum antiviral drug Ribavirin and antiprotozoal drug Dimetridazole could efficiently inhibit the QS system of *P. aeruginosa* and protect *Caenorhabditis elegans* and mice from *P. aeruginosa* infection.

## Materials and methods

### Bacterial strain and media

*Pseudomonas aeruginosa* model strain PAO1 and clinical *P. aeruginosa* strains (SIIA-2 to SIIA-11) from the patients with chronic obstructive pulmonary disease used in this study were previously described elsewhere (Zhao et al., 2018, 2020). The media used were lysogeny broth (LB), Muller Hinton broth (MH), M9 minimum growth medium supplemented with 0.5% casein (w/v), 0.5% (w/v) of skim milk powder, or 0.1% (w/v) of adenosine (Darch et al., 2012; Rezzoagli et al., 2020). Single colony of *P. aeruginosa* PAO1 was inoculated into 5 ml of LB broth and cultured overnight (16–18 h) at 37°C with shaking (220 rpm). Bacterial cells were collected by centrifugation and adjusted to OD_600_ = 1.0 in 1 ml of sterile saline for further use.

### Screening of compounds

Based on the core structure of *P. aeruginosa* QS signals, a total of 40 marketed compounds with poor antibacterial efficacy but have the core structure of furan, benzofuran, or flavonoids were purchased from the MedChemExpress company (Shanghai, China). Equal amount (2 μl) of *P. aeruginosa* PAO1 was inoculated in 200 μl of LB broth or M9-adenosine medium (Darch et al., 2012) supplemented with different concentrations (0, 50, 100, and 200 μM) of the candidate compounds (Supplementary Table S1) and cultured overnight at 37°C. The growth status of *P. aeruginosa* PAO1 was determined by measuring the cell density at OD_600_ using microplate reader (BioTek). To test the susceptibility of *P. aeruginosa* PAO1 to Dimetridazole or Ribavirin, equal amount (10 × 10^5^ CFUs) of *P. aeruginosa* PAO1 was inoculated in 200 μl of MH broth supplemented with 100–4,000 μM of Dimetridazole (corresponds to 14–564 μg/ml) or Ribavirin (corresponds to 24.4–976.8 μg/ml) and cultured for 24 h, followed by measuring the cell densities at OD_600_. All the experiments were independently repeated for three times.

### Proteolysis assay

The production of QS-controlled extracellular proteases of *P. aeruginosa* PAO1 was measured as previously descripted elsewhere (Zhao et al., 2020). Equal amount (2 μl) of *P. aeruginosa* PAO1 was inoculated in 200 μl M9-casein medium with different concentrations (0, 50, 100, and 200 μM) of Dimetridazole or Ribavirin, and cultured overnight at 37°C. Clinical *P. aeruginosa* inoculated in M9-casein medium with 200 μM Dimetridazole or Ribavirin in a similar manner. The growth status of *P. aeruginosa* PAO1 and Clinical *P. aeruginosa* were determined by measuring the cell density at OD_600_ using microplate reader (BioTek). Equal amount (2 μl) of *P. aeruginosa* PAO1 was spotted on M9-skim milk plates with different concentrations (0, 50, 100, and 200 μM) of Dimetridazole or Ribavirin and cultured overnight at 37°C. Clinical *P. aeruginosa* were spotted on M9-skim milk plates with 200 μM Dimetridazole or Ribavirin in a similar manner. The diameter of the proteolytic ring formed around the macrocolony was then measured.

### Biofilm formation assay

The biofilm formation assay of *P. aeruginosa* was carried out according to the method reported by Kim et al. (2021). Briefly, the 96-well plate was used as a medium for biofilm attachment. Equal amount (2 μl) of *P. aeruginosa* PAO1 was inoculated in 200 μl LB broth with different concentrations of Dimetridazole or Ribavirin (0, 50, 100, and 200 μM), and cultured for 24 h at 37°C. Similarly, clinical *P. aeruginosa* were inoculated with 200 μM Dimetridazole or Ribavirin. Subsequently, after the culture liquid was gently removed, the biofilm formed on the wall of the well was washed by sterile saline for three times to remove free bacteria and biofilm. Subsequently, 200 μl of crystal violet (0.1%, w/v) was added to each naturally air-dried well and incubated for 20 min, and then the crystal violet in the well was removed and washed three times. The stained biofilm was dissolved in 200 μl 95% ethanol solution and quantified at OD_595_.

### Pyocyanin production assay

Equal amount (2 μl) of *P. aeruginosa* PAO1 was inoculated in 2 ml of LB broth supplemented with different concentrations of Dimetridazole and Ribavirin (0, 50, 100, and 200 μM) and cultured for 24 h at 37°C. Likewise, clinical *P. aeruginosa* were inoculated in 2 ml of LB broth supplemented with of 200 μM Dimetridazole and Ribavirin. After the cell density was equalized at OD_600_, pyocyanin in the supernatant was extracted and quantified as previously described (Essar et al., 1990). Briefly, chloroform was added to the supernatant at the ratio of 5:3 followed by violently shaking. After standing and stratifying, the lower chloroform extract was collected and added with 0.2 N HCl at the ratio of 3:1 followed by violently shaking. The upper liquid was collected and centrifuged (12,000 rpm, 5 min), and then 200 μl of supernatant was absorbed into 96-well plate and quantitated at OD_520_.

### Molecular docking

Computer-based small molecule-protein interaction analysis mimicking the docking of Dimetridazole or Ribavirin to the three key QS regulatory proteins of *P. aeruginosa* was performed by using the software AutoDock 4 and AutoDockTools according to the producer’s guidelines. The “pdb” files presenting the crystal structure of LasR (3IX3), and PqsR (6B8A) were downloaded from the PubChem database,^1^ and RhlR (P54292) was predicted by AlphaFold in the UniProt.^2^

### Transcriptomic analysis

TRIzol (Thermo) reagents and Total RNA Isolation Kit with gDNA removal (Foregene Biotechnology, Co. Ltd., China) were used to extract the total RNAs of Dimetridazole and Ribavirin (200 μM) treated and untreated *P. aeruginosa* PAO1, respectively. RNA-sequencing (RNA-seq) was accomplished by the Illumina-based HiseqTM2500 platform (Novogene Bioinformatics Technology, China). The data obtained by sequencing were uploaded to the NCBI BioProject database under accession number PRJNA723215. The software Bowtie2-2.2.3 (Langmead and Salzberg, 2012), HTSeq v0.6.1 (Anders et al., 2015), and DESeq 2 (Love et al., 2014) were used to map the filtered reads to *P. aeruginosa* PAO1 and calculate the values of differential gene expression through using expected fragments per kilobase of transcript per million fragments (FPKM). Differentially expressed gene with an adjusted value of *p* (*p*adj) *p* < 0.05 was thought to be significantly different. Enrichment for Kyoto Encyclopedia of Genes and Genomes (KEGG) and Gene Ontology (GO) gene sets was assessed by using clusterProfiler 4.0 (Wu et al., 2021). Differentially expressed QS-regulated genes in *P. aeruginosa* were screened by mapping the genes to previously established list of QS-induced genes (Schuster et al., 2003) by using Venn Diagrams.^3^

### Quantitative PCR

TRIzol reagents were used to extract the total RNAs of Dimetridazole and Ribavirin (200 μM) treated and untreated *P. aeruginosa* PAO1. Quantitative PCR was performed by using an iTaq™ universal SYBR® Green Supermix (Bio-Rad) and the CFX Connect Real-Time PCR Detection System to validate the expression of typical QS-activated genes, including *lasR*, *lasB*, *rhlR*, *rhlA*, *pqsR*, *pqsA*, *pqsD*, *hcnA*, and *phzA* (Supplementary Table S3) using 16S rRNA as the internal reference gene. Gene expression was calculated by the 2^-ΔΔct^ method (Zhao et al., 2018).

### Combining antibiotics with Dimetridazole and Ribavirin assay

The minimum inhibitory concentrations (MICs) of commonly used antibiotics on *P. aeruginosa* PAO1 and clinical isolates were determined by broth dilution method (Humphries et al., 2021). Briefly, the concentrations of experimental strains were adjusted to 1 × 10^5^ CFU/ml with MH broth, and 100 μl of bacterial solution was dispensed into a 96-well plate, followed by the addition of different concentrations of antibiotics, each antibiotic dilution with three biological replicates. After incubating the 96-well plate at 37°C for 16–18 h, the cell density was measured at OD_600_ using microplate reader.

According to the MIC result, isolates SIIA-4, SIIA-9, and SIIA-11 with high resistance to polymyxin B, meropenem, and kanamycin were selected for the antibiotic-antivirulence combination assays. We set up three different concentrations of Dimetridazole and Ribavirin (50, 100, and 200 μM) combined with 13 different concentrations of antibiotics. At the same time, only groups supplemented with different concentrations of Dimetridazole and Ribavirin and groups only supplemented with antibiotics were set as controls. The Bliss independence model was used to evaluate the degree of synergy in growth inhibition for each antibiotic-antivirulence combination (Baeder et al., 2016; Barbosa et al., 2018; Rezzoagli et al., 2020). The results were calculated by the formula of *S* = *f_x,0_·f_0,y_* - *f_,xy_*, where *f_x,0_* was the growth level measured under the antibiotic exposure at concentration *x* when Dimetridazole and Ribavirin concentration was 0; *f_0,y_* was the growth level under Dimetridazole and Ribavirin effect at the *y* concentration when antibiotic concentration was 0; *f_x,y_* was the growth level measured under the combined treatment of *x* and *y* concentrations. If *S* > 0, the two drugs act synergistically. *S* < 0 indicates that the two drugs are antagonistic, and *S* = 0 indicates that the two drugs are independent (Rezzoagli et al., 2020).

### *Caenorhabditis elegans* killing assays

The pathogenicity of *P. aeruginosa* was investigated by fast killing model of *C. elegans*. Equal amount (20 μl) of *P. aeruginosa* PAO1 was smeared on peptone-glucose-sorbitol agar medium with and without Dimetridazole and Ribavirin (200 μM), cultured overnight at 37°C (Kirienko et al., 2014). The plates were then taken out and naturally cooled to room temperature. Subsequently, 10 adult nematodes at L4 stage were seeded on the plates and cultured at 25°C for 96 h. The survival of *C. elegans* in each experimental group was observed and recorded. Nematodes fed with uracil nutrition-deficient *Escherichia coli* OP50 were set as negative control.

### Mice and ethics statement

C57BL/6 mice (8-week-old, female) were bought from the Dossy Experimental Animals Company (Chengdu, China) and housed in a specific-pathogen-free facility at the State Key Laboratory of Biotherapy, Sichuan University. All the animal experiments were approved by the Ethics Committee of the State Key Laboratory of Biotherapy, and carried out in compliance with institutional guidelines concerning animal use and care of Sichuan University.

### Mouse models

Overnight-cultured *P. aeruginosa* PAO1 were collected and diluted to OD_600_ = 0.5 in sterile saline. C57BL/6 female mice were anaesthetized by intraperitoneal injection of ketamine (50 μg ml^−1^) in sterile saline. A total of 1.0 × 10^7^ CFU bacterial cells in 50 μl of sterile saline supplemented with or without Dimetridazole or Ribavirin (200 μΜ) were intranasally instilled into the lungs of mice. Dimetridazole-or Ribavirin-treated mice were then intranasally instilled with 200 μΜ (50 μl) of Dimetridazole or Ribavirin every 12 h, and control group added an equal amount of DMSO. The survival/death status of mice was recorded by observers blinded to the groups. The whole lungs were aseptically removed, and a slice of a pulmonary lobe from each mouse was used for histological examination. Approximately, 0.1–0.2 g of lung tissue was aseptically excised and homogenized in sterile saline for CFU enumeration on King’s B plates.

### Statistical analysis

GraphPad Prism v8.0.1 (San Diego, CA, United States) was used to process the data generated by the phenotypic identification assays. Mean values of SD were compared by using two-tailed unpaired *t* test, or One-way ANOVA test. The survival curves of *C. elegans* and mouse models were compared by using Log-rank (Mantel-Cox) test.

## Results

### Preliminary screening of compounds with anti-QS activity

**Figure 1:**
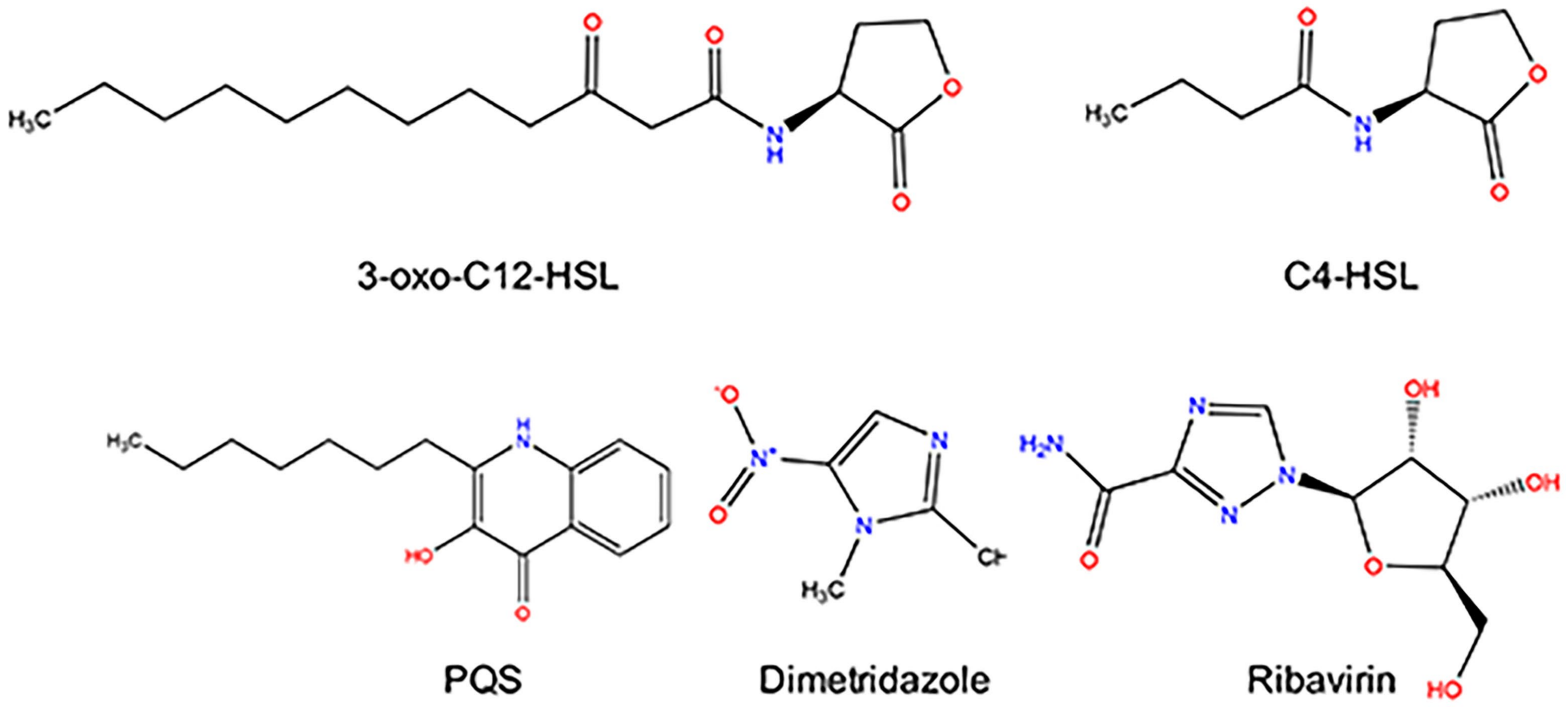
Structure of *Pseudomonas aeruginosa* QS signal molecules (3-oxo-C12-HSL, C4-HSL, and PQS), Dimetridazole, and Ribavirin.

**Figure 2:**
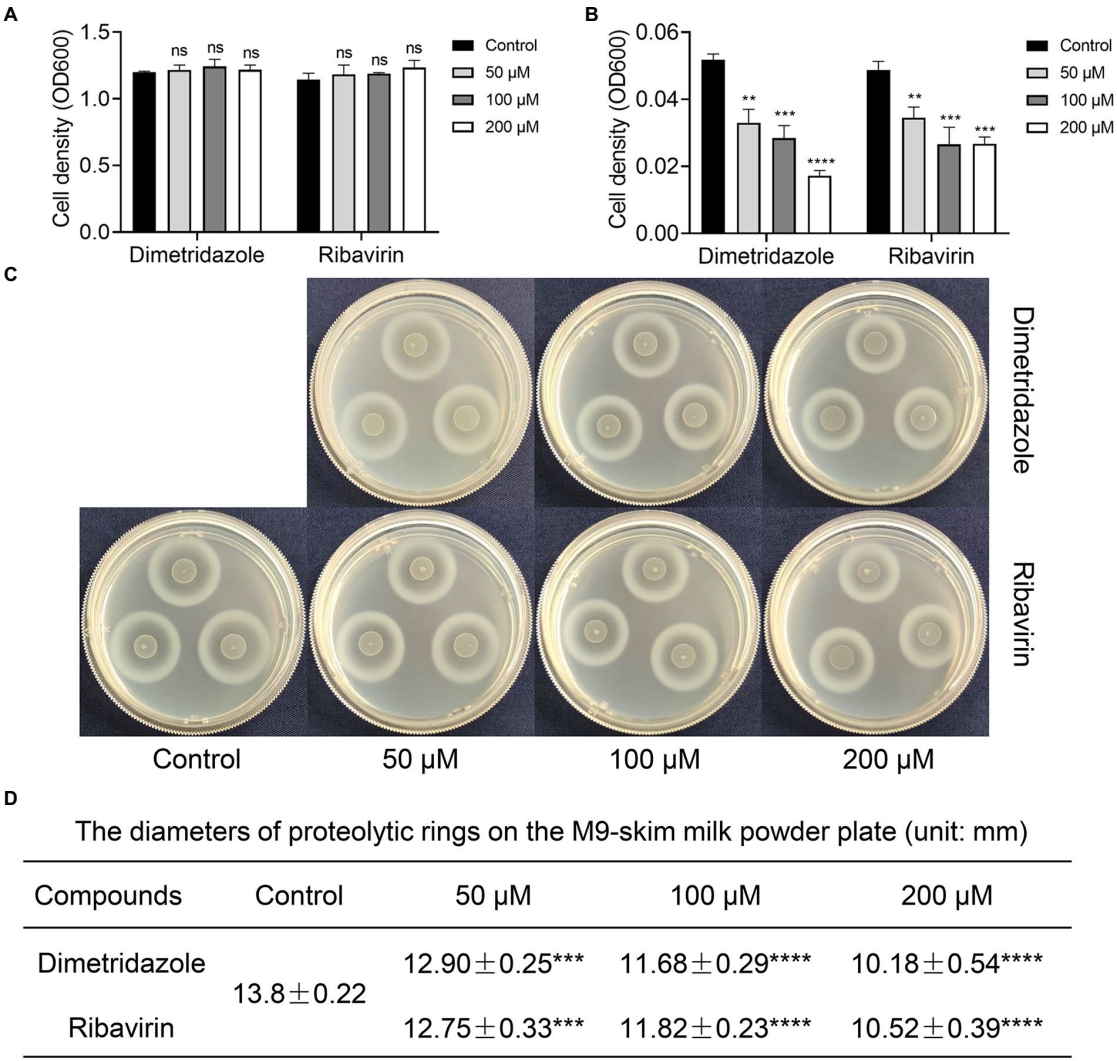
Effects of Dimetridazole and Ribavirin on the growth of *Pseudomonas aeruginosa* PAO1. **(A)** Growth of *P. aeruginosa* PAO1 in LB broth containing different concentrations of Dimetridazole or Ribavirin. **(B)** Growth of *P. aeruginosa* PAO1 in M9-adenosine broth containing different concentrations of Dimetridazole or Ribavirin. **(C)** Growth of *P. aeruginosa* PAO1 on M9-skim milk plate containing different concentrations of Dimetridazole or Ribavirin. **(D)** The diameters of proteolytic rings formed by *P. aeruginosa* PAO1 colony on M9-skim milk plate (unit: mm). Data shown are the means ± SD of at least six independent experiments. One-way ANOVA test compared to the control, **p < 0.01, ***p < 0.001, and ****p < 0.0001. ns, not significant.

Previous study had confirmed that compared to the normal growth of wild-type and QS-deficient *P. aeruginosa* in nutrient medium, the growth of *P. aeruginosa* in M9 minimal growth medium supplemented with adenosine as the sole carbon source requires the QS-induced purine nucleosidase Nuh (Darch et al., 2012). In this study, *P. aeruginosa* PAO1 was first cultured in LB and M9-adenosine broth to preliminarily screen the compounds (100 μM) with poor antibacterial activity but remarkable QS inhibition activity. Finally, Dimetridazole and Ribavirin, which have similar core structure to the AHL signals (Figure 1), were obtained (Supplementary Table S1). We further found that Dimetridazole and Ribavirin had no significant effect on the growth of *P. aeruginosa* PAO1 in LB or MH broth when their concentrations were lower than 800 μM (112 μg/ml) and 1,000 μM (244.2 μg/ml), respectively, but showed a dose-dependent growth inhibition effect in M9-adensine (Figures 2A,B and Supplementary Figure S1). These results suggested that Dimetridazole and Ribavirin at routinely used concentrations had poor antibacterial activity on *P. aeruginosa* PAO1, but might inhibit the QS system. Therefore, Dimetridazole and Ribavirin were selected to further study their antivirulence activities.

### Dimetridazole and Ribavirin inhibit the virulence-related phenotypes of *Pseudomonas aeruginosa*

**Figure 3:**
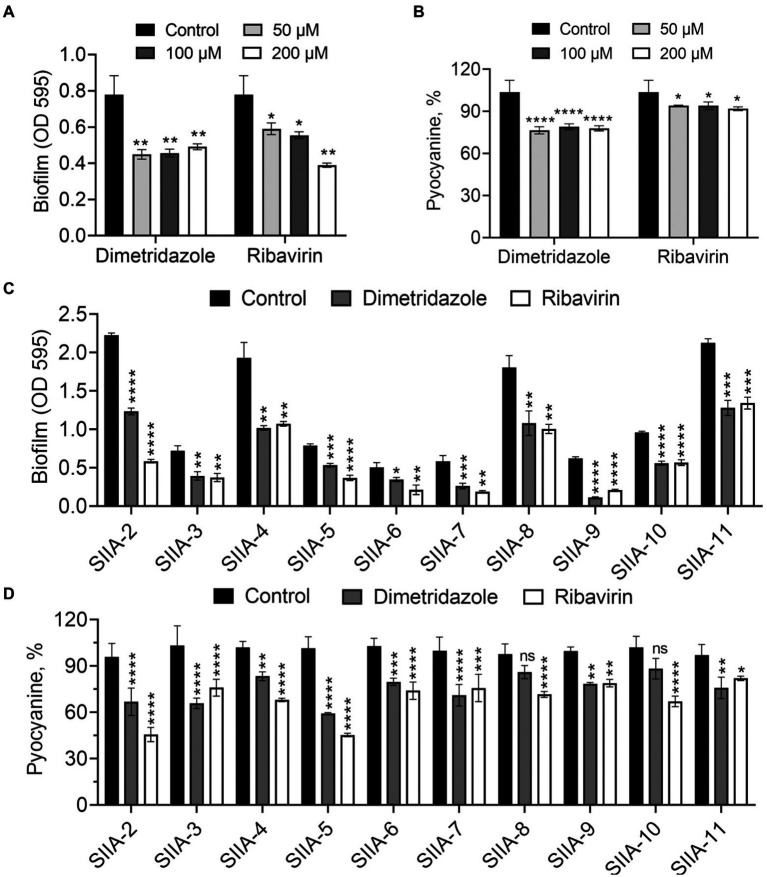
Effects of Dimetridazole and Ribavirin on biofilm and pyocyanine production of *Pseudomonas aeruginosa*. **(A,B)** Effects of different concentrations of Dimetridazole and Ribavirin on the production of biofilm and pyocyanin in *P. aeruginosa* PAO1. **(C,D)** Effects of Dimetridazole and Ribavirin (200 μM) on biofilm and pyocyanine production of *P. aeruginosa* clinical isolates. Data shown are means ± SD of three independent experiments. One-way ANOVA test compared to the control, *p < 0.05, **p < 0.01, ***p < 0.001, ****p < 0.0001. ns, not significant.

Growth of *P. aeruginosa* in QS-required media such as M9-skim milk and in M9-casein are dependent of the production of QS-controlled extracellular proteases (e.g., the virulence factor elastase; Sandoz et al., 2007). In this study, the antivirulence effects of Dimetridazole and Ribavirin on *P. aeruginosa* were evaluated by measuring the growth status and the size of proteolytic ring produced by the colony in QS-required media. Compared to the apparent proteolytic ring formed by untreated colony, the addition of Dimetridazole or Ribavirin significantly reduced the production of extracellular proteases by *P. aeruginosa* PAO1 in a dose-dependent manner, as well as the growth in M9-casein broth (Figures 2C,D and Supplementary Figure S2A). Similar results were observed in clinical *P. aeruginosa* isolates with an intact ability to produce extracellular proteases (SIIA-4, SIIA-8, SIIA-10, and SIIA-11; Supplementary Figures S2B, S3, S4). We also assessed the influences of Dimetridazole and Ribavirin on the production of other virulence factors in *P. aeruginosa*, such as pyocyanin and biofilm. The results showed that both the production of pyocyanin and biofilm of *P. aeruginosa* PAO1 and the tested clinical isolates, including the isolates deficient in producing extracellular proteases, were significantly decreased upon the treatment of Dimetridazole or Ribavirin (Figure 3). These results clearly demonstrated the efficient antivirulence activities of Ribavirin and Dimetridazole on *P. aeruginosa* isolates.

### Docking of Dimetridazole and Ribavirin to *Pseudomonas aeruginosa* QS regulators

Computer-based small molecule-protein interaction analysis was performed to mimic the docking of Dimetridazole or Ribavirin to the three key QS regulatory proteins of *P. aeruginosa*. Among the six experimentally confirmed binding sites (Tyr-56/Trp-60/Arg-61/Asp-73/Thr-75/Ser-129) of native 3-oxo-C12-HSL to LasR (Bottomley et al., 2007), 3-oxo-C12-HSL might simultaneously bind to Tyr-56, Trp-60, and Ser-129 locating in the AHL-binding pocket of LasR (Supplementary Figure S5A). By contrast, Dimetridazole and Ribavirin might tightly bind to Arg-61/Ser-129 and Asp-73/Ser-129 in the ligand-binding domain (LBD) of LasR, respectively (Supplementary Figures S5B,C). The native C4-HSL might bind to the Tyr-72 and Ser-135 sites of RhlR LBD (Supplementary Figure S6A). Although Dimetridazole might be capable of docking to the AHL-binding pocket of RhlR, it has no interaction with the predicted active site (Supplementary Figure S6B). Differently, Ribavirin might bind to the Tyr-72 site of RhlR LBD (Supplementary Figure S6C). Furthermore, both Dimetridazole and Ribavirin might be capable of binding to the position similar to the binding of native PQS to PqsR (Supplementary Figure S7). These data preliminarily showed that Dimetridazole and Ribavirin might have the potential to simultaneously influence the regulations of LasR, RhlR, and PqsR in *P. aeruginosa*.

### Effects of Dimetridazole and Ribavirin on the global transcription of *Pseudomonas aeruginosa*

**Figure 4:**
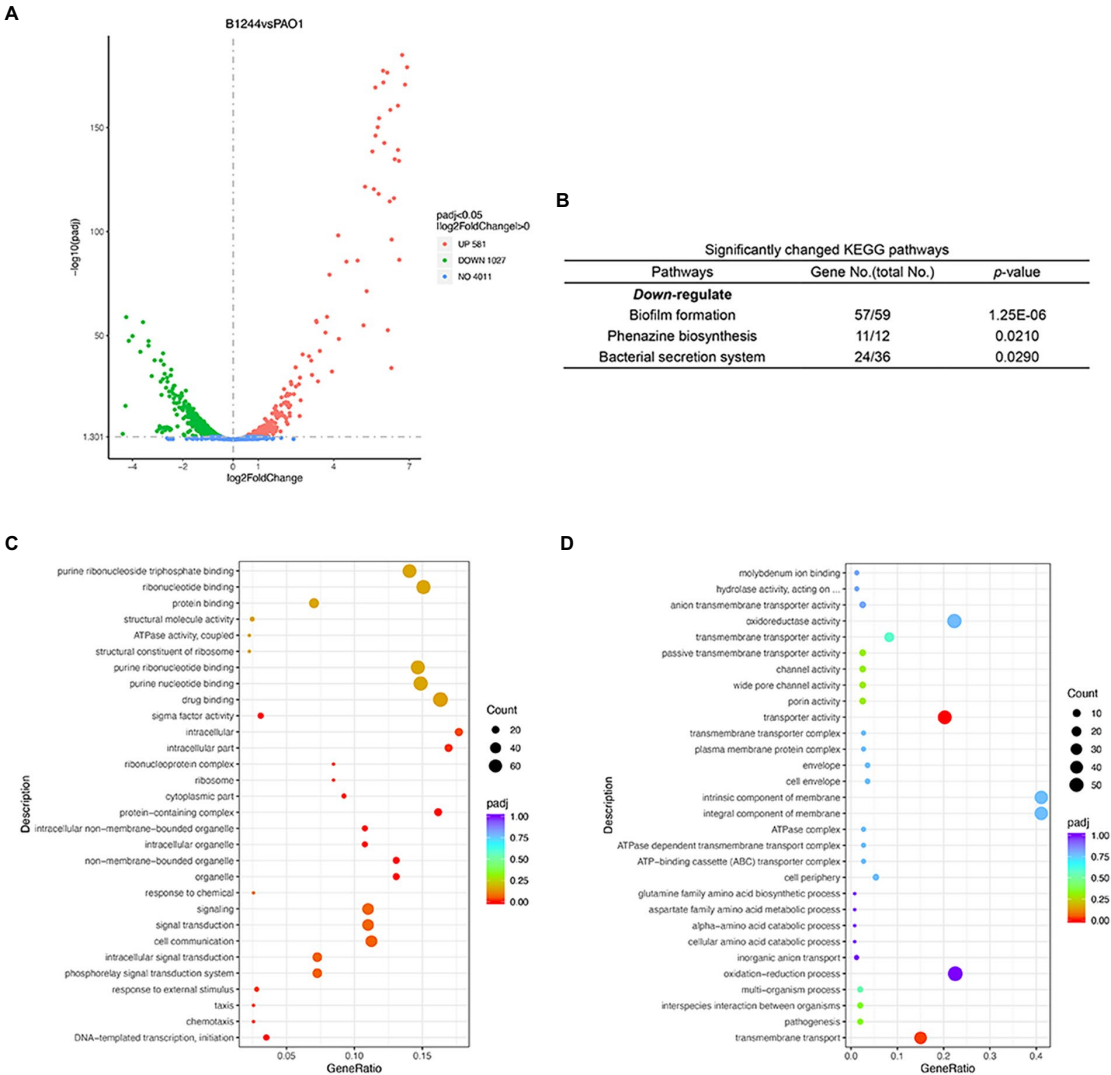
Effects of Dimetridazole on the transcriptome of *Pseudomonas aeruginosa* PAO1. **(A)** Number of significantly differentially expressed genes of *P. aeruginosa* PAO1 influenced by 200 μM of Dimetridazole. **(B)** Significantly changed KEGG terms influenced by 200 μM of Dimetridazole. Significantly **(C)** downregulated and **(D)** upregulated GO terms caused by 200 μM of Dimetridazole.

**Figure 5:**
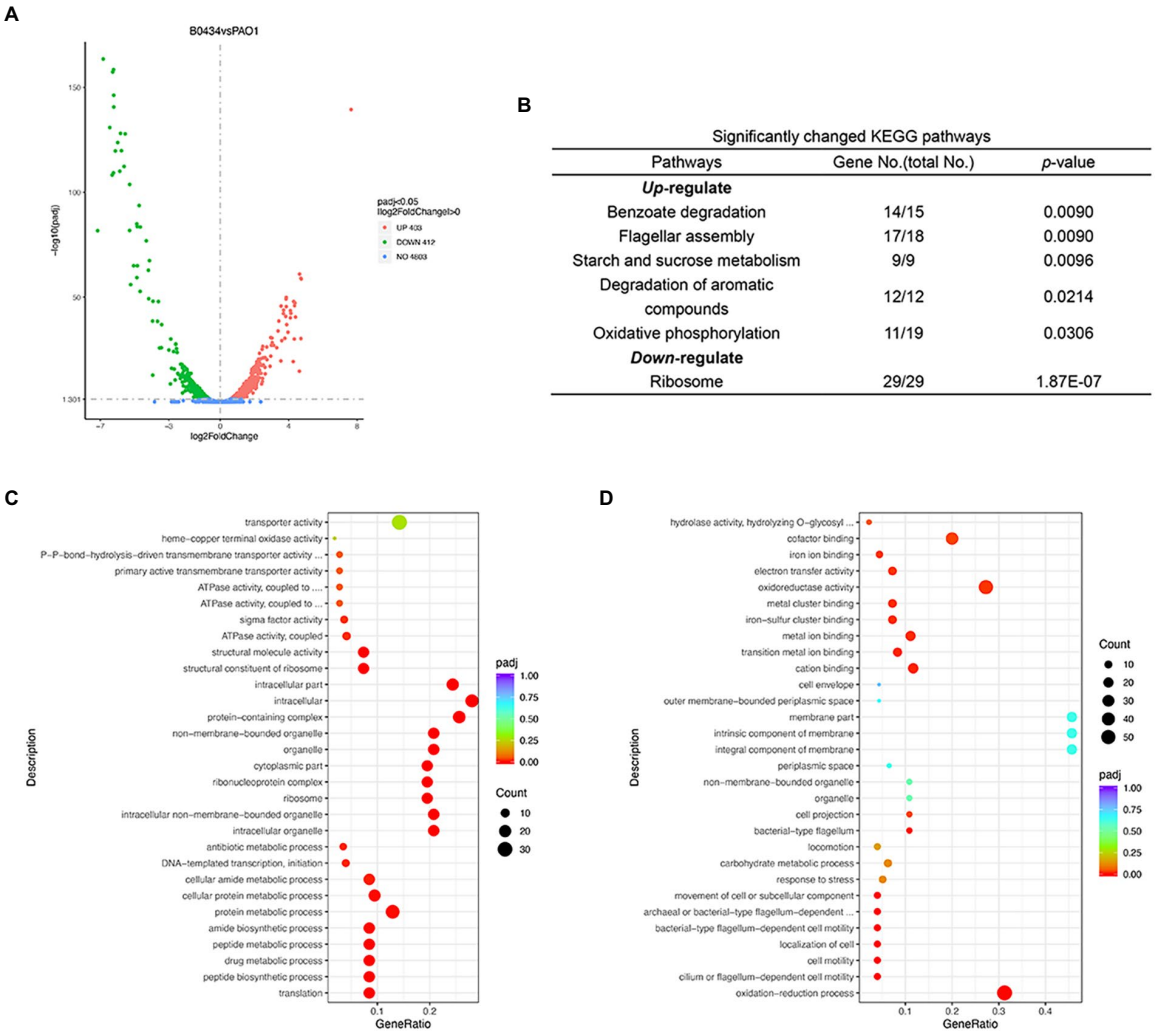
Effects of Ribavirin on the transcriptome of *Pseudomonas aeruginosa* PAO1. **(A)** Number of significantly differentially expressed genes of *P. aeruginosa* PAO1 influenced by 200 μM of Ribavirin. **(B)** Significantly changed KEGG terms influenced by 200 μM of Ribavirin. Significantly **(C)** downregulated and **(D)** upregulated GO terms caused by 200 μM of Ribavirin.

The changes in the transcriptome of *P. aeruginosa* PAO1 caused by Dimetridazole or Ribavirin treatment were investigated by RNA-seq. Compared to the control group, 581 upregulated genes and 1,207 downregulated (*p* < 0.05) were identified in *P. aeruginosa* PAO1 treated with 200 μM of Dimetridazole (Figure 4A; Supplementary Dataset 1). KEGG functional annotation suggested that the biofilm formation, bacterial secretion system, and phenazine biosynthesis of Dimetridazole-treated *P. aeruginosa* PAO1 were significantly enriched (*p* < 0.05) by the down-regulated genes (Figure 4B). Moreover, the GO terms of DNA-templated transcription and initiation, chemotaxis, organelle, protein-containing complex, ribosome, intracellular part, and sigma factor activity were significantly decreased in Dimetridazole-treated *P. aeruginosa* PAO1 (Figure 4C), while the transporter activity was increased (Figure 4D). In Ribavirin treatment group, 403 genes were upregulated and 412 genes downregulated (Figure 5A and Supplementary Dataset 2). The KEGG terms of benzoate degradation, degradation of aromatic compounds, flagellar assembly, etc., were considerably enriched (*p* < 0.05) among the upregulated genes in *P. aeruginosa* PAO1 treated by Ribavirin, while ribosome was the sole KEGG term significantly enriched by the downregulated genes (Figure 5B). In the enrichment of GO terms, Dimetridazole significantly decreased the drug metabolic process, protein metabolic process, antibiotic metabolic process, aerobic respiration, ribosome, intracellular part, ATPase activity, and sigma factor activity, but increased the oxidation–reduction process, cell motility, and metal ion binding of *P. aeruginosa* PAO1 (Figures 5C,D). These results suggested that the global transcription, especially the virulence and secondary metabolism-related functions of *P. aeruginosa*, was significantly influenced upon the treatment of Dimetridazole or Ribavirin.

### Dimetridazole and Ribavirin inhibit the QS regulation of *Pseudomonas aeruginosa*

**Figure 6:**
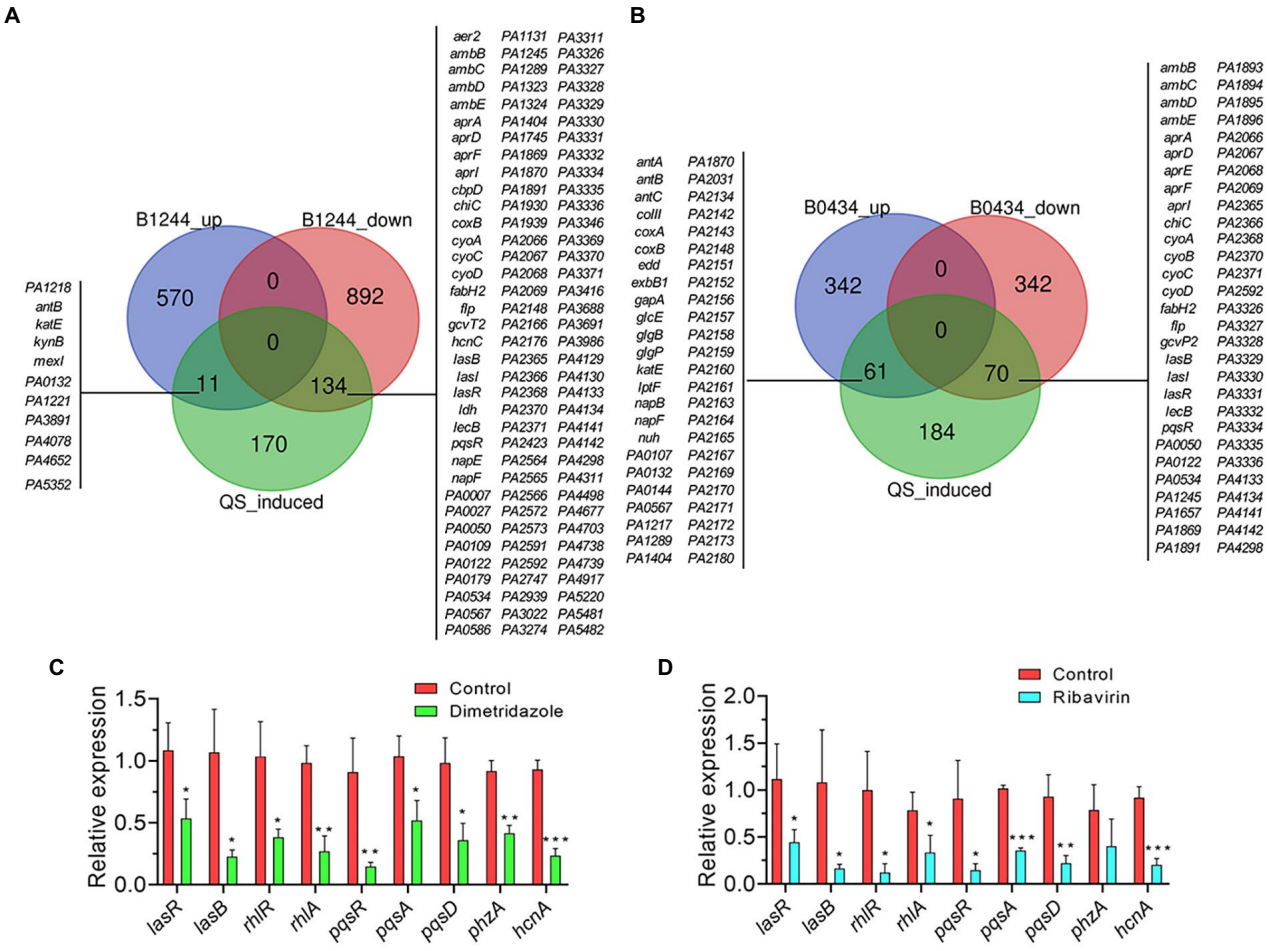
Effects of Dimetridazole and Ribavirin on the expression of QS-induced genes in *Pseudomonas aeruginosa* PAO1. **(A)** Number and list of significantly differentially expressed QS-induced genes in Dimetridazole-treated *P. aeruginosa* PAO1. **(B)** Number and list of significantly differentially expressed QS-induced genes in Ribavirin-treated *P. aeruginosa* PAO1. B1244, Dimetridazole. B0434, Ribavirin. Expression of typical QS-induced genes in **(C)** Dimetridazole-and **(D)** Ribavirin-treated *P. aeruginosa* PAO1 as determined by quantitative PCR. Data shown are the means ± SD of three independent experiments. Unpaired two-tailed *t* test, *p < 0.05, **p < 0.01, ***p < 0.001.

To further understand the effects of Dimetridazole and Ribavirin on the expression of QS-related genes in *P. aeruginosa* PAO1, all the significantly changed genes were applied to the list of QS-induced genes previously released by Schuster et al. (2003). The results showed that compared to the untreated control, the expression levels of 134 and 70 genes positively controlled by *P. aeruginosa* QS system were significantly decreased by the presence of Dimetridazole and Ribavirin, respectively (Figures 6A,B). The results of quantitative PCR further confirmed that the expression levels of typical QS-induced genes in *P. aeruginosa* PAO1, including the regulatory genes *lasR*, *rhlR*, and *pqsR* and their downstream genes (*lasB*, *rhlA*, *pqsA*, *pqsD*, *phzA*, and *hcnA*), were inhibited by Dimetridazole or Ribavirin by 2–6 folds (Figures 6C,D). Therefore, these data combined with the phenotypic identification above (Figures 2, 3), collectively confirmed the inhibitory effects of Dimetridazole and Ribavirin on the QS regulation of *P. aeruginosa*.

### Dimetridazole and Ribavirin increase the susceptibility of *Pseudomonas aeruginosa* to antibiotics

**Figure 7:**
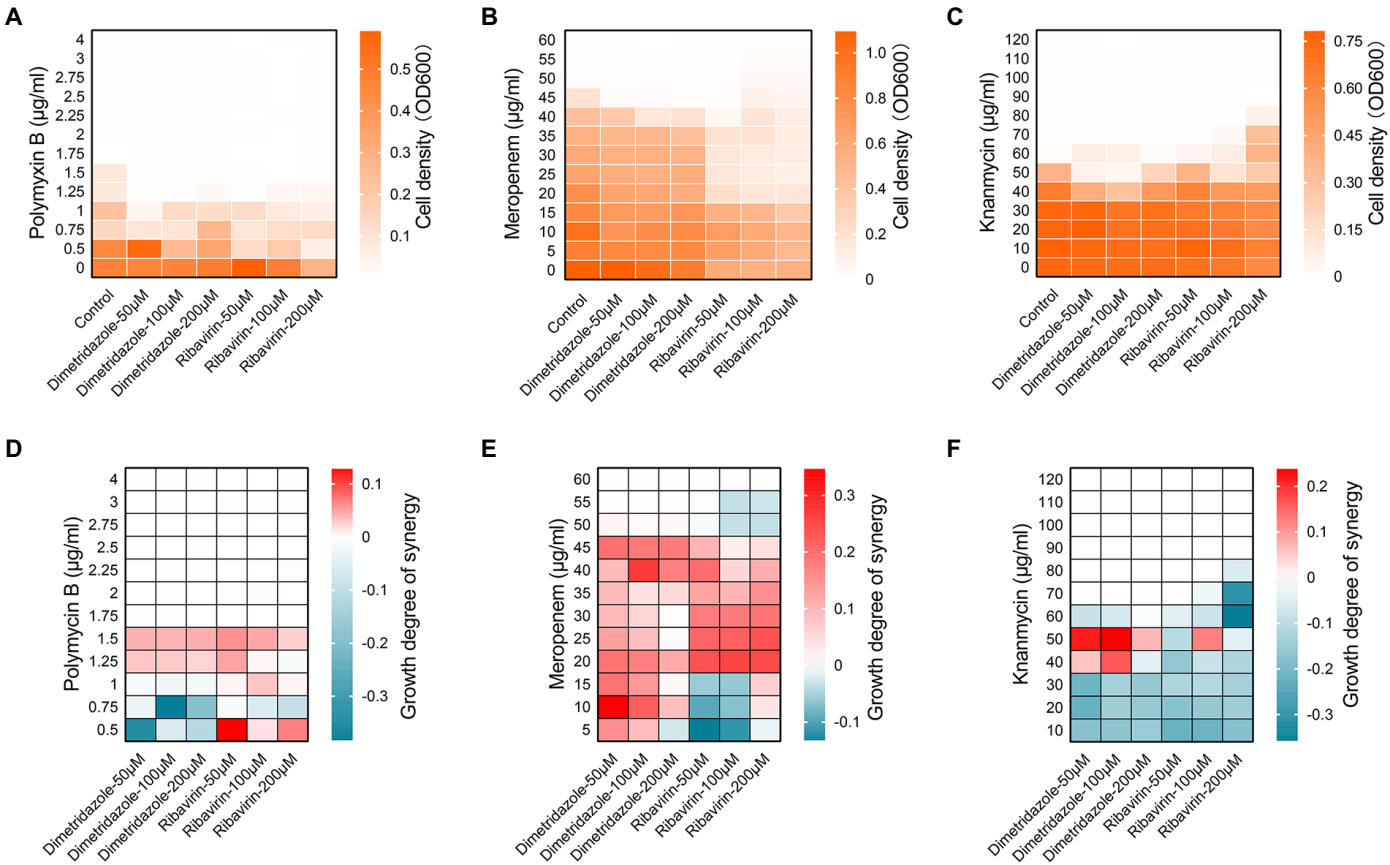
Synergic interactions of Dimetridazole and Ribavirin with antibiotics. Cell densities of *Pseudomonas aeruginosa* clinical isolates **(A)** SIIA-4, **(B)** SIIA-9, and **(C)** SIIA-11 cultured in MH broth supplemented with Dimetridazole or Ribavirin and polymyxin B, meropenem, or kanamycin at different combinations. Influences of different combinations of Dimetridazole or Ribavirin and polymyxin B, meropenem, or kanamycin on the growth of **(D)** SIIA-4, **(E)** SIIA-9, and **(F)** SIIA-11 as calculated by the Bliss independence mode. Tiffany blue color indicates antagonistic interaction. Red color indicates synergistic interaction.

We then set out to explore the synergistic interactions of Dimetridazole and Ribavirin with commonly used antibiotics in combating the drug-resistant *P. aeruginosa* isolates. The results showed that the susceptibility of *P. aeruginosa* clinical isolates to polymyxin B, meropenem, or kanamycin was promoted by the supplementation of Dimetridazole or Ribavirin, albeit the effective concentrations might be varied among groups (Figures 7A–C and Supplementary Table S2). Specifically, the addition of Dimetridazole or Ribavirin at the concentrations of 50–200 μM significantly increased the susceptibility of *P. aeruginosa* clinical isolate SIIA-4 to polymyxin B (Figure 7A). Differently, Ribavirin showed constant synergistic interactions with polymyxin B at all the tested concentrations as determined by the synergistic analysis using Bliss model (Baeder et al., 2016), while synergistic interactions of Dimetridazole and polymyxin B were detected only when the concentration of polymyxin B was higher than 1 μg/ml (Figure 7D). The addition of Dimetridazole reduced the cell density of meropenem-resistant *P. aeruginosa* isolate SIIA-9 to meropenem, and showed constant synergistic interactions with meropenem at all the tested concentrations (Figures 7B,E). By contrast, the supplementation of Ribavirin resulted in a remarkable decrease in the cell density of SIIA-9 compared to the culture solely treated by meropenem, and stable synergistic interactions of Ribavirin and meropenem were detected when the concentration of meropenem was ranged from 20–45 μg/ml (Figures 7B,E). Finally, the synergistic interaction of Dimetridazole and kanamycin in inhibiting the growth of kanamycin-resistant isolate SIIA-11 was detected only when the concentration of kanamycin was ranged from 40–50 μg/ml, while Ribavirin showed constant antagonistic effects with kanamycin (Figures 7C,F). Therefore, these results revealed the potentials of Dimetridazole and Ribavirin to promote the antibacterial efficiency of antibiotics in a manner of synergistic interaction.

### Dimetridazole and Ribavirin protect *Caenorhabditis elegans* and mice from *Pseudomonas aeruginosa* infection

**Figure 8:**
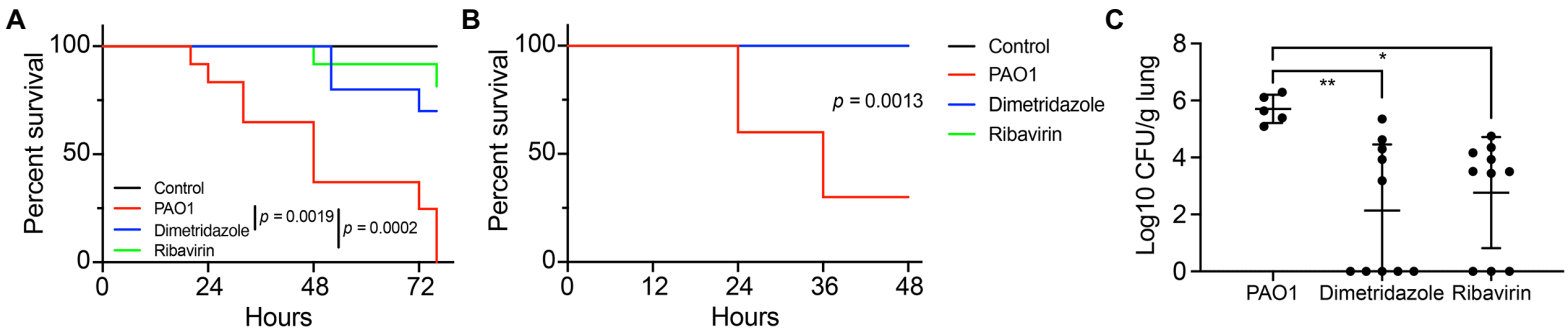
Dimetridazole or Ribavirin effectively protects the host from *Pseudomonas aeruginosa* infection. **(A)** Protection of Dimetridazole or Ribavirin (200 μM) on *Caenorhabditis elegans* models infected with *P. aeruginosa* PAO1 in the fast killing assay. **(B)** Protection of Dimetridazole or Ribavirin (200 μM) on mouse models intranasally instilled with *P. aeruginosa* PAO1. The survival curves were compared by using Log-rank (Mantel-Cox) test. **(C)** Number of residual *P. aeruginosa* PAO1 cells in mouse lungs treated or not treated with Dimetridazole or Ribavirin. One-way ANOVA test, *p < 0.05, **p < 0.01.

We then investigated the *in vivo* antivirulence activities of Dimetridazole and Ribavirin on *P. aeruginosa* PAO1 by using *C. elegans* and mouse models. The results showed that the supplementation of Dimetridazole (*p* = 0.0019) or Ribavirin (*p* = 0.0002) significantly slowed down the killing of *C. elegans* by *P. aeruginosa* PAO1 compared to the untreated group (Figure 8A). Similarly, compared to the fast killing of mice by *P. aeruginosa* PAO1 during acute lung infection, all the mice were survived in the group treated with Dimetridazole or Ribavirin (*p* = 0.0013), and the residual bacterial CFUs in mouse lungs were significantly lower than the untreated group, or even completely removed (Figures 8B,C). Therefore, these results suggested that the treatment of Dimetridazole or Ribavirin effectively protected *C. elegans* and mice from acute *P. aeruginosa* infection.

## Discussion

Antibiotic resistance of pathogenic microorganisms are turning into an increasing global crisis, especially in the declining amount of new and effective antibiotic development today (Munguia and Nizet, 2017; Hegazy et al., 2020; Imperi et al., 2021). *Pseudomonas aeruginosa* is one of the most notorious and intractable human pathogens because of its complex regulatory network in causing a wide range of infections (Lee and Zhang, 2015; Valentini et al., 2018; Mukherjee and Bassler, 2019). *Pseudomonas aeruginosa* has a variety of antibiotic resistance mechanisms, which usually result in the failure of clinical treatment (Boucher et al., 2009). As a consequence, new alternative strategies against *P. aeruginosa* infection are becoming increasingly urgent. In the present study, we identify the antivirulence activities of the antizoan drug Dimetridazole and antiviral drug Ribavirin on *P. aeruginosa* PAO1 and the clinical isolates by inhibiting the QS system.

In comparison with the traditional antibiotics, antivirulence drugs will not kill or halt the growth of bacterial pathogens in nutrient media (Kalia and Purohit, 2011). The QS system of *P. aeruginosa* controls the production of several virulence factors and is closely related to the formation of bacterial biofilms (Balasubramanian et al., 2013; Zhao et al., 2019). AHL-dependent QS mutants exhibit reduced lethality in a variety of animal models (Tang et al., 1996; Pearson et al., 2000). These evidences provide a direction for researchers to develop novel anti-pseudomonal drugs by targeting the central regulator/s QS system (Kalia and Purohit, 2011; Fleitas Martínez et al., 2019). By testing the antibacterial and antivirulence potentials of known compounds with similar core structure to the AHL signals of *P. aeruginosa* QS system but are not commonly used for anti-infective therapy, here we find that Dimetridazole and Ribavirin had poor inhibitory effect on the growth of *P. aeruginosa* in LB broth but inhibited the virulence of *P. aeruginosa in vitro* and *in vivo*. O’Loughlin et al. (2013) reported that synthetic meta-bromo-thiolactone (mBTL) inhibited the production of pyocyanin and biofilm formation of *P. aeruginosa* by targeting the QS receptors LasR and RhlR (O’Loughlin et al., 2013). In the context of drug repurposing, sub-inhibitory cefepime, ceftazidime, and ceftriaxone were found to work as QS inhibitors to inhibit the motility, pyocyanin biosynthesis, and biofilm formation of *P. aeruginosa*, and also enhance the antibacterial effect of aminoglycoside antibiotics (Kumar et al., 2021). As a treatment for diabetes, sitagliptin was also found to have QS inhibitory activity (Hegazy et al., 2020). Therefore, in addition to chemosynthesis and functional screening, our present study provides an alternative strategy for the discovery of antivirulence drugs by directly screening candidate compounds from marketed drugs based on the core structure of AHL signals, and this may greatly facilitate the development of novel anti-infective agents for clinical application.

Clinical evidence has suggested that *P. aeruginosa* isolates with kinds of loss-of-function mutations in *lasR* gene but highly active *rhlR* regulon are frequent in the lungs of patients with cystic fibrosis (Feltner et al., 2016). The work by Chen et al. (2019) clearly confirmed that *P. aeruginosa lasR* mutants are also capable of producing QS-regulated extracellular products through *rhlR* and *pqsR* signaling (Chen et al., 2019). These evidences indicate that the development of antivirulence strategy targeting a sole QS regulator may lead to the failure of clinical therapy. Correspondingly, many compounds (such as metformin, and 6-gingerol.) with obvious QS inhibition activity *in vitro* failed to protect mice from *P. aeruginosa* infection (Kim et al., 2015; Hegazy et al., 2020). The result of molecular docking in the present study revealed that both Dimetridazole and Ribavirin may bind to the active sites or dock to the LBD of LasR, RhlR, and PqsR (Supplementary Figures S5–S7). This prediction combined with the decreased the expression of a large set of QS-induced genes determined by RNA-seq and qPCR verification, and the 100% of survival rate of mice (Figures 6, 8), collectively demonstrate that Dimetridazole and Ribavirin may serve as promising antivirulence compounds simultaneously targeting the three core regulators of *P. aeruginosa* QS system.

Furthermore, combined application of drugs has become a common anti-infection method in clinic, which aims to retain the therapeutic effect of the drugs and to prevent the prevalence of bacterial resistance (Lehár et al., 2009; Foucquier and Guedj, 2015; Duarte and Vale, 2022). It is reported that colistin and tobramycin with the antivirulence compounds gallium and furanone C-30 effectively reduced pyoverdine production and antibiotic resistance of *P. aeruginosa* (Rezzoagli et al., 2020). Additionally, baicalin was found to effectively inhibit the biofilm formation of *P. aeruginosa* and enhance the growth inhibition activities of antibiotics (Luo et al., 2017). In this study, in addition to the antivirulence activities of Dimetridazole and Ribavirin, we further identified the synergistic interactions of them with polymyxin B, meropenem, and kanamycin to promote the susceptibilities of *P. aeruginosa* clinical isolates that are highly resistant to corresponding antibiotics (Figure 7). These findings uncover another possible application of Dimetridazole and Ribavirin for the treatment of antibiotic-resistant *P. aeruginosa*-related infections with combined use of common antibiotics.

Overall, this study reveals the antivirulence activities of Dimetridazole and Ribavirin and their synergistic interactions with antibiotics against *P. aeruginosa*. The supplementation of Dimetridazole or Ribavirin inhibits in the production of *P. aeruginosa* virulence factors including pyocyanin, extracellular proteases, and biofilm, promotes the susceptibilities of *P. aeruginosa* to antibiotics, and significantly protects *C. elegans* and mice from the challenge of *P. aeruginosa*. Therefore, this study provides new perspective and structural basis for the development of effective antivirulence drugs for the treatment of *P. aeruginosa* infection, and contributes to addressing the looming public health dilemma of the emergence of multidrug-resistant bacteria.

## Data availability statement

The datasets presented in this study can be found in online repositories. The names of the repository/repositories and accession number(s) can be found in the article/Supplementary material.

## Ethics statement

The animal study was reviewed and approved by The Ethics Committee of the State Key Laboratory of Biotherapy, Sichuan University.

## Author contributions

KZ designed the research. YY, XY, QZ, and RF performed the experiments. KZ and LD performed the bioinformatic analyses. HL and XZo coordinated the mice infection experiments. YZ, WL, YC, and XZa provided critical experimental equipment and materials. KZ, YY, and XZa analyzed data and wrote the manuscript. All authors contributed to the article and approved the submitted version.

## Funding

This work was supported by the National Natural Science Foundation of China (31970131, 81922042, and 82172285), Sichuan Science and Technology Program (2021JDJQ0042), 1·3·5 project of excellent development of discipline of West China Hospital of Sichuan University (ZYYC21001), and innovation foundation of the Affiliated Hospital of Chengdu University (CDFYCX202209).

## Conflict of interest

The authors declare that the research was conducted in the absence of any commercial or financial relationships that could be construed as a potential conflict of interest.

## Publisher’s note

All claims expressed in this article are solely those of the authors and do not necessarily represent those of their affiliated organizations, or those of the publisher, the editors and the reviewers. Any product that may be evaluated in this article, or claim that may be made by its manufacturer, is not guaranteed or endorsed by the publisher.

## Footnotes

^1^ https://pubchem.ncbi.nlm.nih.gov

^2^ https://www.uniprot.org

^3^ http://bioinformatics.psb.ugent.be/webtools/Venn/
