# Supplementary material for: Repurposing Dimetridazole and Ribavirin to disarm Pseudomonas aeruginosa virulence by targeting the quorum sensing system
Source: Front Microbiol. 2022 Aug 15;13:978502. doi: 10.3389/fmicb.2022.978502 (PMC9421001; doi:10.3389/fmicb.2022.978502)
Supplement: Supplementary file 1 [file Data_Sheet_1.PDF]

# Repurposing Dimetridazole and Ribavirin to Disarm *Pseudomonas aeruginosa* Virulence by Targeting the Quorum Sensing System

**Yang Yuan[1,2], Xiting Yang[2], Qianglin Zeng[2], Heyue Li[1], Ruyi Fu[2], Lianming Du[2], Wei Liu[2], Yamei Zhang[2], Xikun Zhou[3], Yiwen Chu[2], Xiuyue Zhang[1,*] and Kelei Zhao[2,*]**

[1] Key Laboratory of Bio-resources and Eco-environment, Ministry of Education, College of Life Sciences, Sichuan University, Chengdu 610064, Sichuan, China.

[2] Antibiotics Research and Re-evaluation Key Laboratory of Sichuan Province, School of Pharmacy, Affiliated Hospital/Clinical College of Chengdu University, Chengdu University, Chengdu 610106, Sichuan, China.

[3] State Key Laboratory of Biotherapy and Cancer Center, West China Hospital, West China Medical School, Sichuan University, and Collaborative Innovation Center for Biotherapy, Chengdu 610041, Sichuan, China.

*** Correspondence:**

Kelei Zhao, address: No. 2025, Chengluo Avenue, Chengdu 610051, Sichuan, China. Email: zhaokelei@cdu.edu.cn.

Xiuyue Zhang, address: No. 24, South Section 1, Yihuan Road, Chengdu 610064, Sichuan, China. Email: zhangxiuyue@scu.edu.cn.

## Supplementary Figures

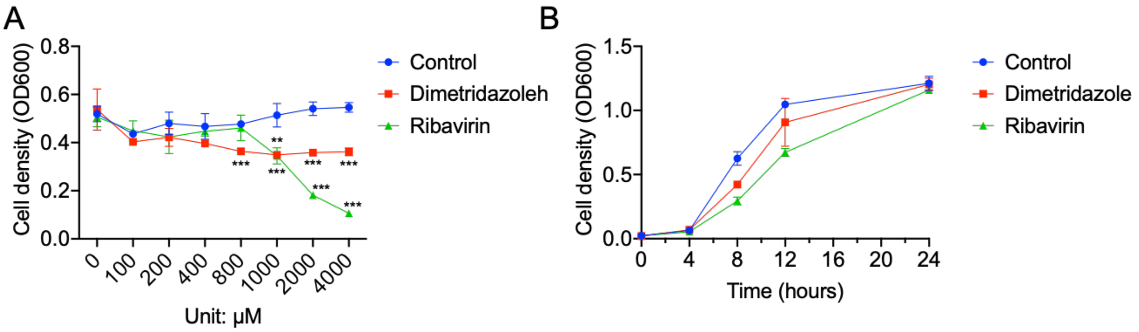


**Supplementary Figure S1.** Susceptibility of *Pseudomonas aeruginosa* PAO1 to Dimetridazole and Ribavirin. **(A)** Dose-dependent influences of Dimetridazole or Ribavirin on the growth of *P. aeruginosa* PAO1. Equal amount (10 × $10^5$ CFUs) of *P. aeruginosa* PAO1 was inoculated in 200 μL of Mueller-Hinton (MH) broth supplemented with 100–4000 μM of Dimetridazole (corresponds to 14–564 μg/mL) or Ribavirin (corresponds to 24.4–976.8 μg/mL) and cultured for 24 h. Data shown are means ± standard deviation (SD) of three independent replicates. One-way ANOVA test compared to the control, ** $p < 0.01$, *** $p < 0.001$. Asterisk below the symbol, Dimetridazole. Asterisk above the symbol, Ribavirin. **(B)** Time-dependent influences of 200 μM of Dimetridazole or Ribavirin on the growth of *P. aeruginosa* PAO1. Equal amount (10 × $10^7$ CFUs) of *P. aeruginosa* PAO1 was inoculated in 200 μL of LB broth supplemented with 200 μM of Dimetridazole or Ribavirin. Data shown are means ± SD of three independent replicates.

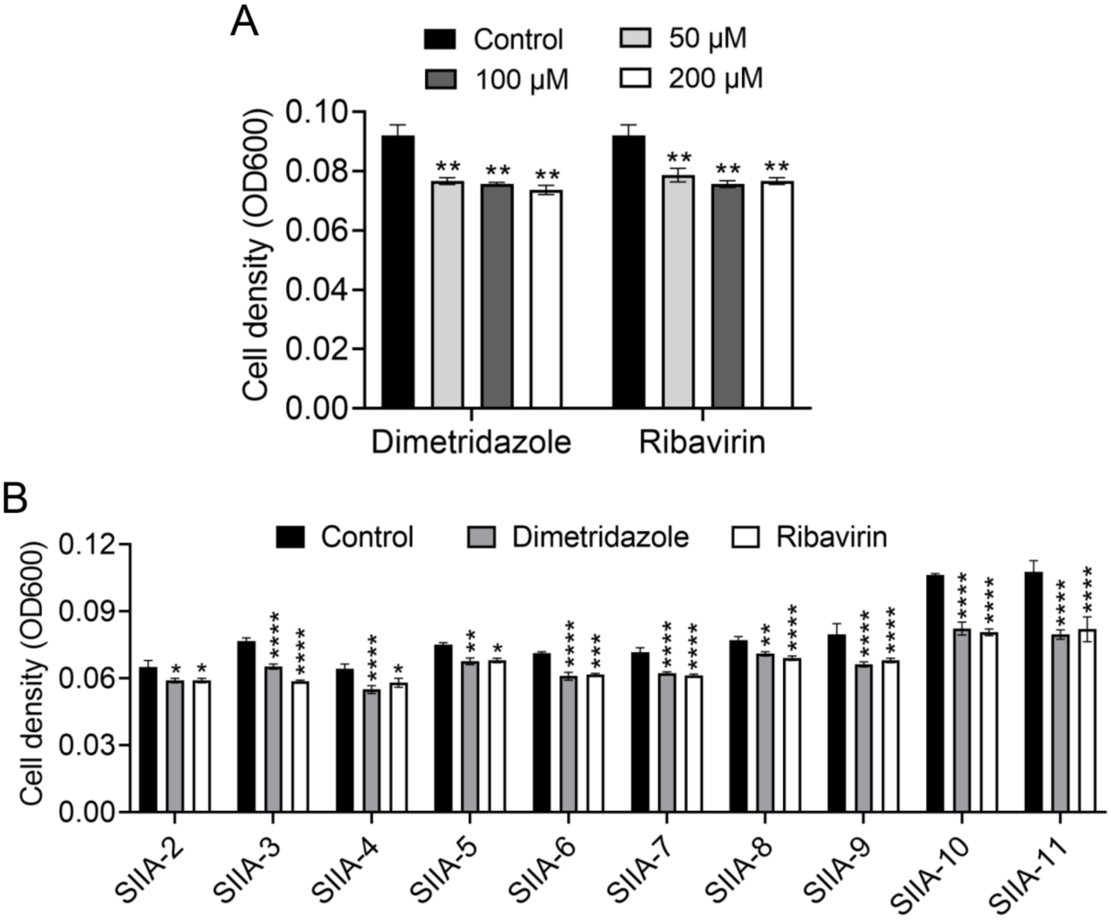


**Supplementary Figure S2.** Effects of Dimetridazole and Ribavirin on the growth of *P. aeruginosa*. A. Growth of *P. aeruginosa* PAO1 in M9-casein broth containing different concentrations of Dimetridazole or Ribavirin. B. Growth of clinical *P. aeruginosa* in M9-casein broth containing with 200 μM Dimetridazole or Ribavirin. Data shown are means ± SD of three independent replicates. One-way ANOVA test compared to the control, * $p < 0.05$, ** $p < 0.01$, *** $p < 0.001$, **** $p < 0.0001$. ns, not significant.

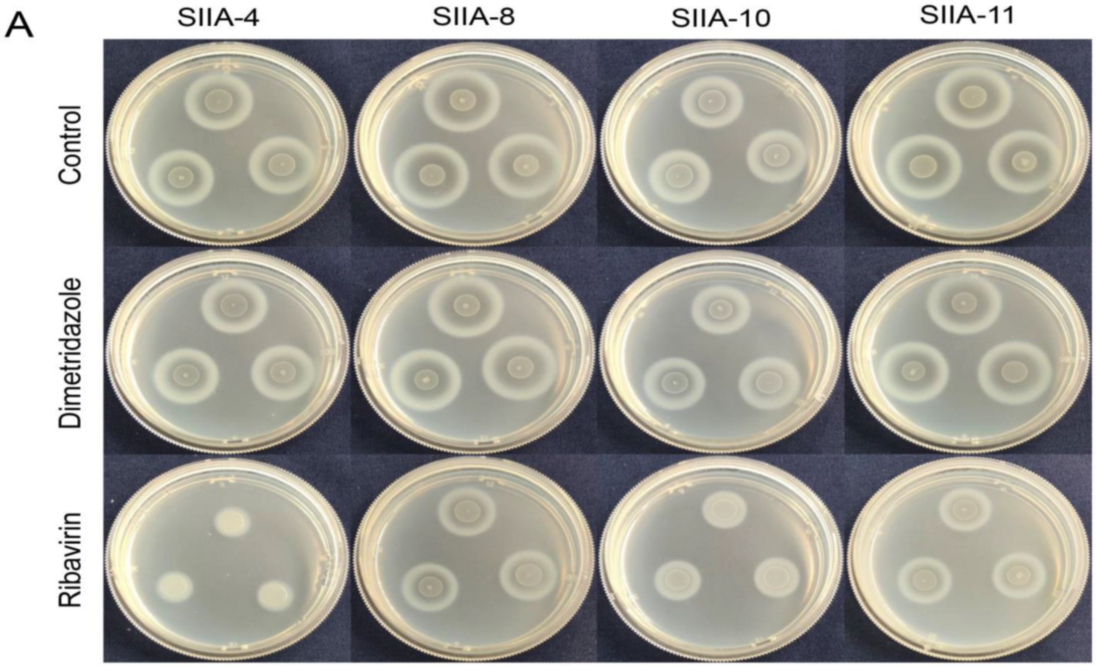


B

The diameters of proteolytic rings in the M9-skim milk powder plate (unit: mm)

| Clinical *P. aeruginosa* | Control | Dimetridazole (200 μM) | Ribavirin (200 μM) |
|---|---|---|---|
| SIIA-4 | 11.02±0.57 | 9.92±0.33** | 0 |
| SIIA-8 | 13.25±0.19 | 12.17±0.39*** | 9.40±0.38**** |
| SIIA-10 | 9.02±0.12 | 7.85±0.21**** | 0 |
| SIIA-11 | 13.33±0.37 | 12.40±0.3** | 8.32±0.26**** |

**Supplementary Figure S3.** Effects of Dimetridazole and Ribavirin on the growth of clinial *P. aeruginosa*. A. Growth of clinical *P. aeruginosa* on M9-skim milk plate containing with 200 μM Dimetridazole or Ribavirin. B. The diameters of proteolytic rings formed by clinial *P. aeruginosa* colony on M9-skim milk plate (unit: mm). Data shown are means ± SD of at least six independent replicates. One-way ANOVA test compared to the control, * $p < 0.05$, ** $p < 0.01$, *** $p < 0.001$, **** $p < 0.0001$. ns, not significant.

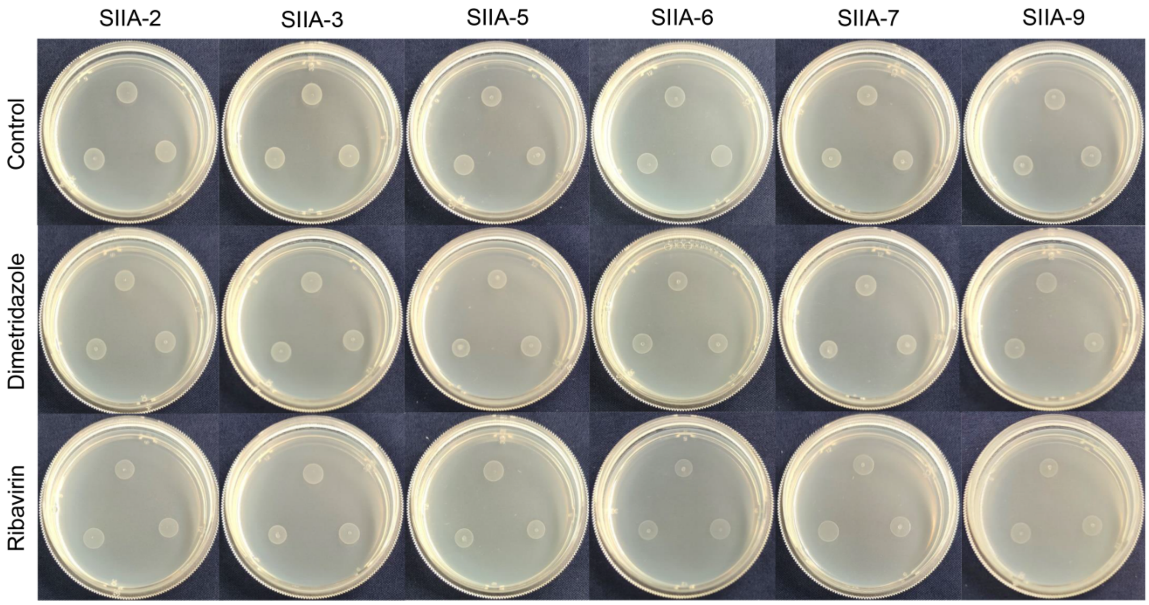


**Supplementary Figure S4.** Growth of clinical *P. aeruginosa* on M9-skim milk plate with 200 μM Dimetridazole or Ribavirin. None of the six clinical *P. aeruginosa* form proteolytic rings.

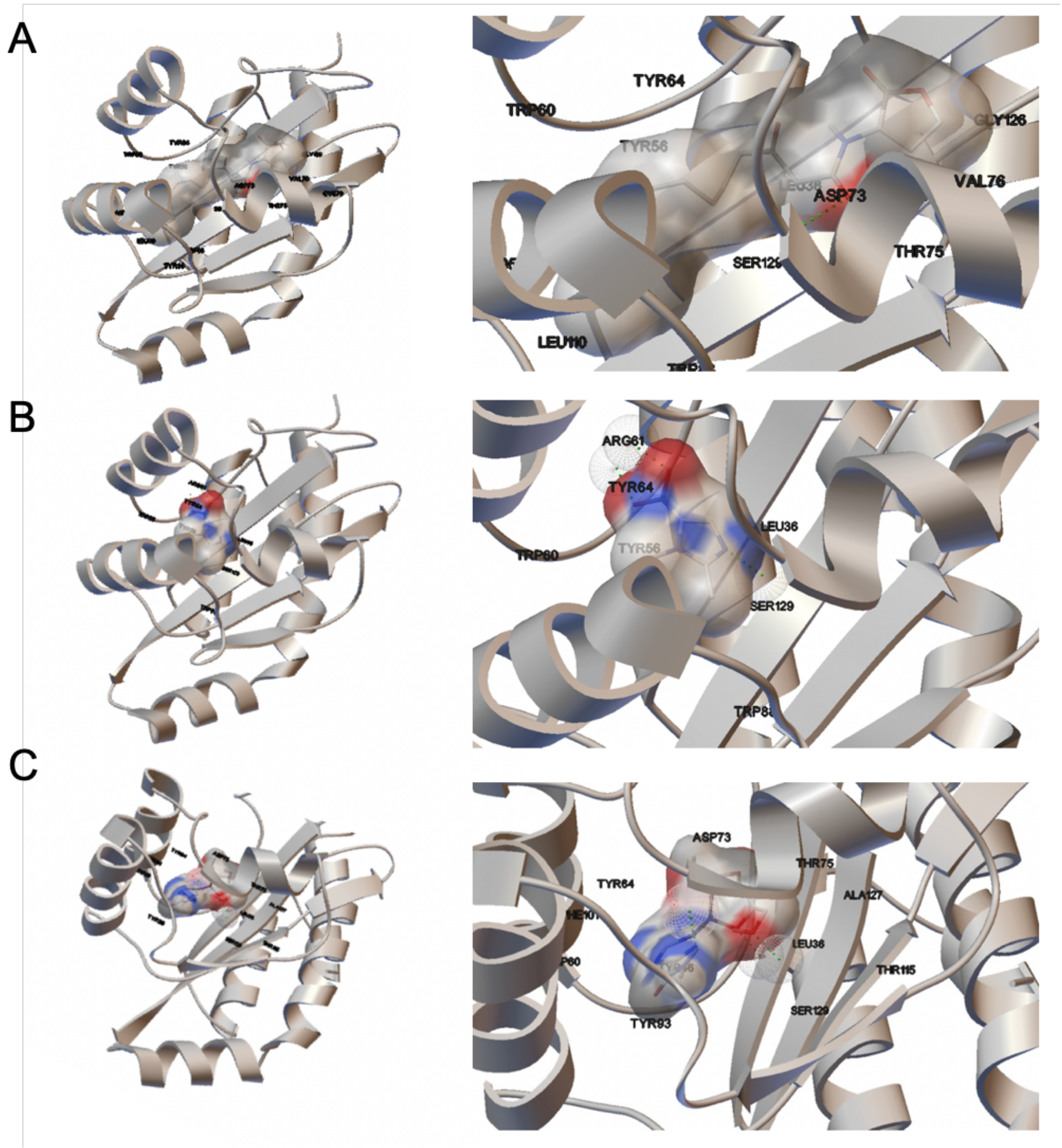


**Supplementary Figure S5.** Molecular docking of (**A**) native C12-HSL signal, (**B**) Dimetridazole, and (**C**) Ribavirin to LasR protein of *P. aeruginosa* PAO1.

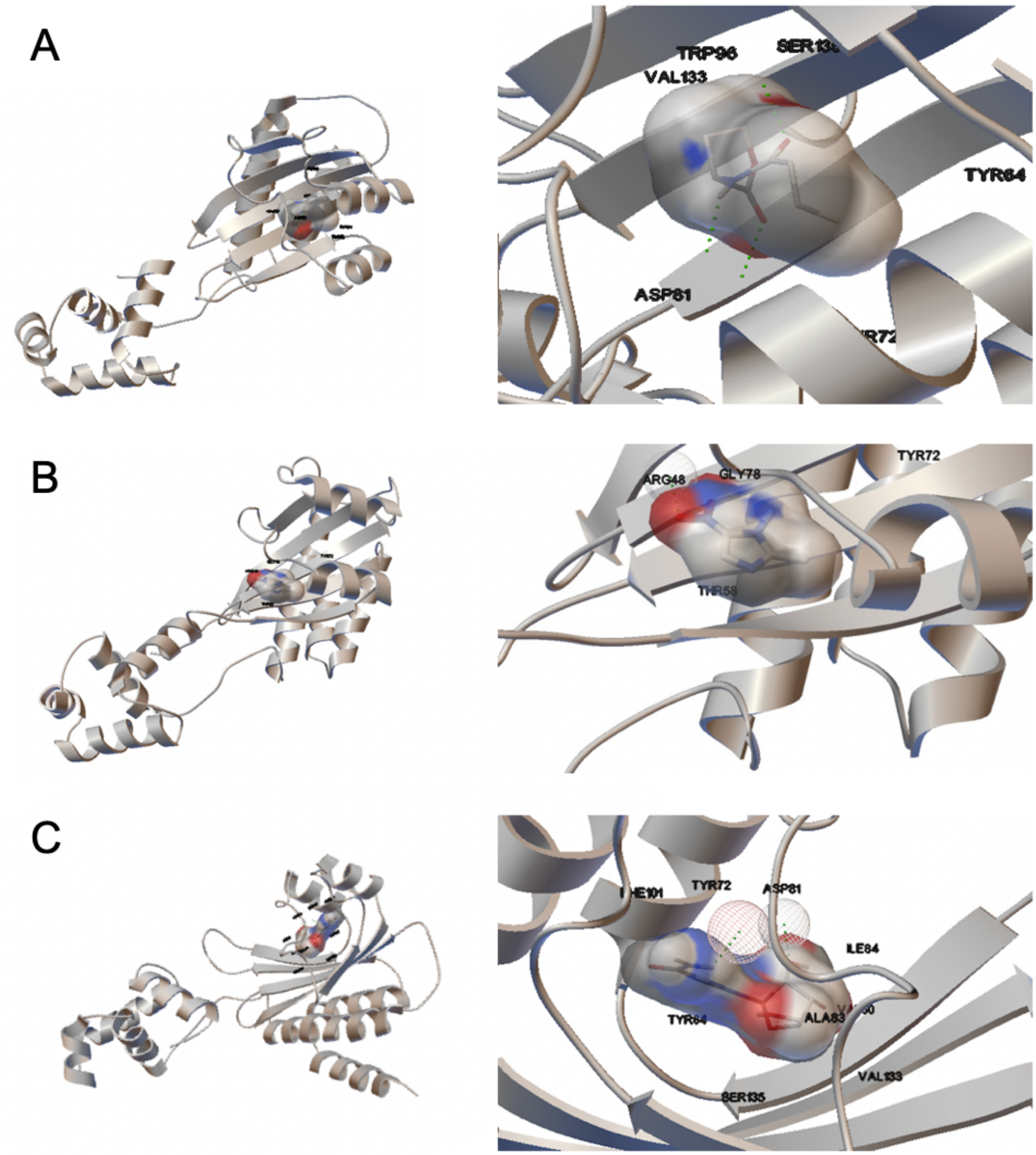


**Supplementary Figure S6.** Molecular docking of (**A**) native C4-HSL signal, (**B**) Dimetridazole, and (**C**) Ribavirin to RhlR protein of *P. aeruginosa* PAO1.

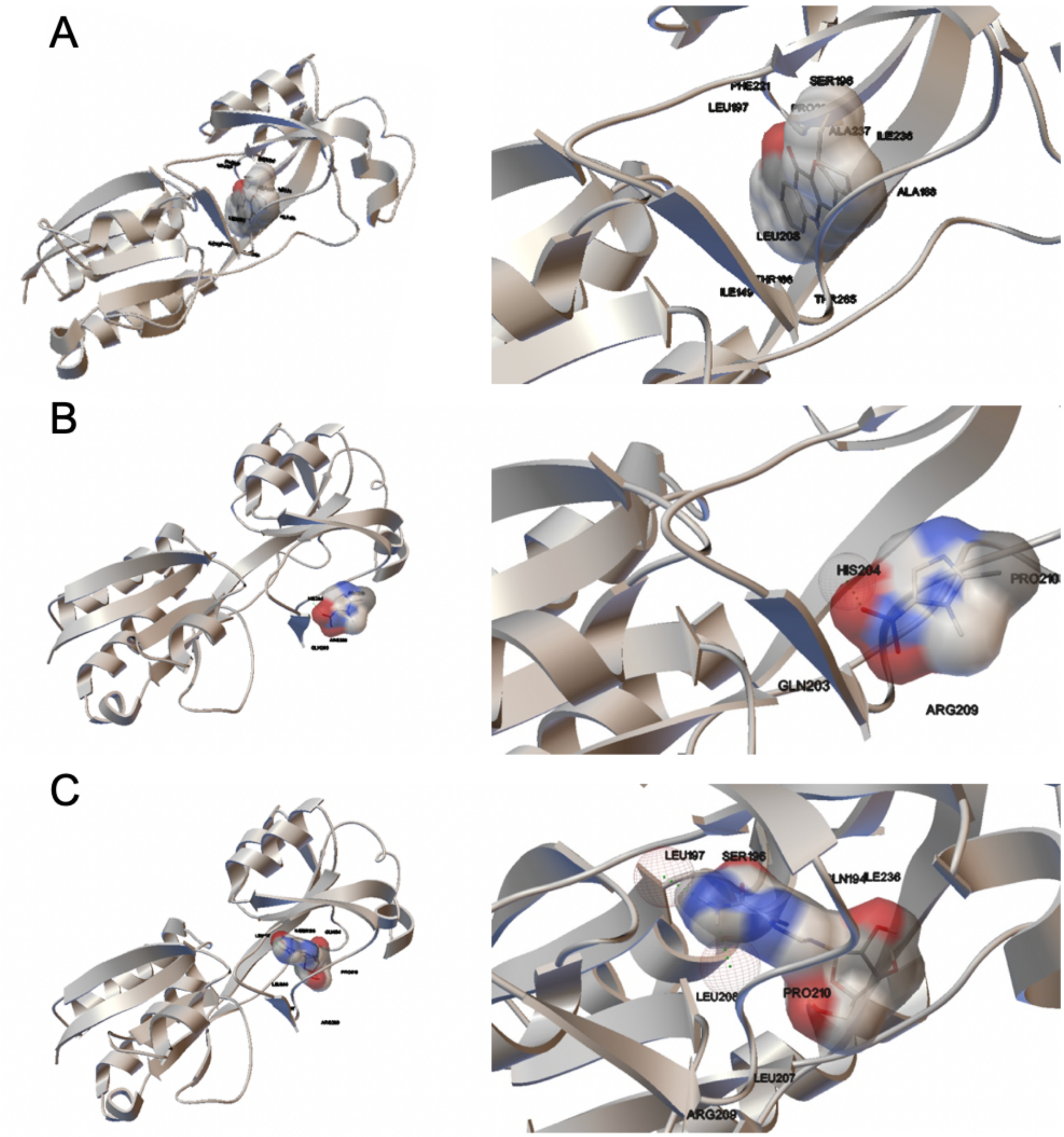


**Supplementary Figure S7.** Molecular docking of (**A**) native PQS signal, (**B**) Dimetridazole, and (**C**) Ribavirin to PqsR protein of *P. aeruginosa* PAO1.

## Supplementary Tables

**Supplementary Table S1.** Effect of 40 compounds on the growth of *P. aeruginosa* PAO1.

| Compounds | Effect of growth[a] |
|---|---|
| 2,5-Dihydroxyphenylacetic acid lactone | Increase |
| 5-Hydroxymethylfurfural | No change |
| Sesamol | Decrease |
| 2-Benzoxazolinone | No change |
| Carbendazim | Increase |
| L-5-Hydroxytryptophan | Increase |
| Fenbendazole | No change |
| 5-Hydroxymethyl-2-furancarboxylic acid | No change |
| DL-3-Phenyllactic acid | No change |
| Urocanic acid | No change |
| L-Pyroglutamic acid | Increase |
| Succinylsulfathiazole | No change |
| Tazobactam | No change |
| 5-Methyluridine | No change |
| Acadesine phosphate | Decrease |
| 2'-Deoxycytidine | Increase |
| 2'-Deoxyguanosine | Decrease |
| Kinetin (6-Furfuryladenine) | No change |
| Cotinine | No change |
| Creatinine | Increase |
| Oxaceprol (N-Acetyl-L-hydroxyproline) | No change |
| Pilocarpine Hydrochloride | Increase |
| Metronidazole | No change |
| Nifursol | Increase |
| Nithiamide | Decrease |
| Furazolidone | No change |
| Nitrofurantoin | Decrease |
| Nitrofurazone | Decrease |
| **Ribavirin** | **Decrease** |
| **Dimetridazole** | **Decrease** |
| Dinotefuran | Increase |
| Ronidazole | Decrease |
| Tizoxanide | Increase |
| Bisantrene | Increase |
| Andrographolide | Increase |

| | |
|---|---|
| Bilobalide | Decrease |
| 5-Amino-3H-imidazole-4-Carboxamide | Increase |
| Allantoin | Increase |
| 5-Methylfurfural | Increase |

*P. aeruginosa* PAO1 were cultured in M9 minimum growth medium supplemented with 0.1% (w/v) of adenosine broth and one kind of compound (100 μM) overnight at 37 ℃. Each experiment was independently repeated for three times.

[a] Compared with *P. aeruginosa* PAO1 treated by DMSO or water, un-paired *t* test, $p < 0.05$. Bold color indicates $p < 0.001$.

**Supplementary Table S2.** Sensitivity of *P. aeruginosa* PAO1and clinical *P. aeruginosa* to commonly used antibiotics (minimal inhibitory concentration, μg/mL).

| *P. aeruginosa* isolates | Polymyxin B | Meropenem | Kanamycin | Aztreonam | Levofloxacin | Tobramycin |
|---|---|---|---|---|---|---|
| PAO1 | 2 | 3 | 16 | 4 | 0.4 | 0.6 |
| SIIA-2 | 2 | 1 | 64 | 2 | 1 | 0.6 |
| SIIA-3 | 2 | 1 | 128 | 8 | 0.4 | 0.6 |
| SIIA-4 | 2 | 32 | 64 | 32 | 0.4 | 0.4 |
| SIIA-5 | 2 | 2 | 128 | 32 | 0.6 | 0.4 |
| SIIA-6 | 2 | 2 | 64 | 8 | 0.8 | 0.4 |
| SIIA-7 | 2 | 3 | 32 | 8 | 1 | 0.6 |
| SIIA-2 | 2 | 1 | 64 | 8 | 0.6 | 0.6 |
| SIIA-8 | 1 | 64 | 64 | 32 | 1 | 0.4 |
| SIIA-9 | 1 | 32 | 128 | 8 | 1 | 0.6 |
| SIIA-10 | 1 | 1 | 128 | 32 | 0.4 | 0.4 |
| SIIA-11 | 2 | 3 | 16 | 4 | 0.4 | 0.6 |

**Supplementary Table S3.** Primers used in this study

| Gene | Sequence (5'-3') |
|---|---|
| *lasR*rtF | CTTCATCGTCGGCAACTAC |
| *lasR*rtR | GTCTGGTAGATGGACGGTTC |
| *lasB*rtF | ATCGGCTACGACATCAAGAAGG |
| *lasB*rtR | CCGCTGTTGTAGTTGCTGGTG |
| *rhlR*rtF | GCTCCTCGGAAATGGTGGT |
| *rhlR*rtR | GGAAAGCACGCTGAGCAAAT |
| *rhlA*rtF | ACTGAACCAGGCGATGCTC |
| *rhlA*rtR | GCTCCAGGCAAGCCAAGTA |
| *pqsR*rtF | CACTGGTTGAAGCGGGAGA |
| *pqsR*rtR | TCGTTCTGCGATACGGTGAG |
| *pqsA*rtF | GCTGAGCGGTCCTTTGGC |
| *pqsA*rtR | TGGAACCCGAGGTGTATTGC |
| *pqsD*rtF | GCTGTACGGCTTGCAGATGG |
| *pqsD*rtR | CAGGTCCAGCAGTCCGTCTT |
| *phzA1rt*F | GCAACTGGACCACGGAAAG |
| *phzA1rt*R | GCACGCAGTTTCTGTATCGG |
| *hcnArt*F | GCAGACATGACCATCCACCTC |
| *hcnArt*R | CGGTTGCTTTCGGTTTCCA |
| *16Srt*F | TCGCATCCTGTTGTCCTCCA |
| *16Srt*R | TTAGCCAGGGTCAGCGTCA |

## Supplementary Datasets

**Supplementary Dataset 1.** Significantly differentially expressed genes of Dimetridazole-treated *Pseudomonas aeruginosa* PAO1. $p$adj < 0.05.
**Supplementary Dataset 2.** Significantly differentially expressed genes of Ribavirin-treated *Pseudomonas aeruginosa* PAO1. $p$adj < 0.05.
